# Screening for substance use and mental health problems in a cross-sectoral sample of Canadian youth

**DOI:** 10.1186/s13033-017-0128-4

**Published:** 2017-02-28

**Authors:** Joanna L. Henderson, Gloria Chaim, Lisa D. Hawke, National Youth Screening Project Network

**Affiliations:** 1 Centre for Addiction and Mental Health, 80 Workman Way, Toronto, ON M6J 1H4 Canada; 2 University of Toronto, 250 College Street, Toronto, ON M5T 1R8 USA

**Keywords:** Youth, Cross-sectoral, Mental health, Screening, Substance use

## Abstract

**Background:**

This project examines the substance use and mental health concerns of a cross-sectoral, national, service-seeking sample of adolescents and emerging adults using an extended version of the Global Appraisal of Individual Needs-Short Screener (GSS). It also aims to provide incremental evidence of the psychometric properties of the GSS.

**Methods:**

A sample of 2313 youth aged 12–24 years who presented for service participated in the project. Youth were recruited from 89 participating services across Canada representing eight major clinical and non-clinical sectors. Participants completed the GSS and provided sociodemographic data.

**Results:**

The majority of youth presenting for services endorsed concerns on the GSS and would be likely to meet diagnostic criteria for a disorder in a full diagnostic assessment according to the norms for the scale, while many endorsed multiple concerns. This was true in both clinical and non-clinical settings. Externalizing concerns and suicidality were significantly more common in younger participants, while substance use was significantly more common in older youth. Females were more likely to endorse internalizing and suicidality concerns, while males endorsed more substance use and crime/violence concerns. Internalizing and suicidality concerns were also more common in Canada’s northerly regions. The reliability of the GSS was confirmed, however the factor structure revealed problems.

**Conclusions:**

Youth presenting across clinical and non-clinical service sectors endorse high levels of need, supporting the importance of universal, cross-sectoral screening. The GSS is a practical tool that service providers across sectors can employ to identify the addiction and mental health service needs of youth, although further psychometric work is warranted. Implications for screening and treatment in community contexts are discussed.

## Background

The majority of mental health disorders (70%) begin in childhood or adolescence [1]. In Canada, an estimated one in five young people are experiencing at least one significant substance use or mental health issue, and suicide is the second leading cause of death among youth [2–4]. Concurrent disorders (CDs; i.e., the co-occurrence of mental health and substance use disorders) are particularly concerning, since CDs are associated with greater severity of disorder, poorer prognosis, increased treatment challenges and greater unmet need for treatment compared to mental health or substance use disorders alone [5–8].

Most children and youth with a mental health disorder, including substance use disorders, do not receive mental health treatment, despite the poor outcomes and high costs associated with untreated mental health problems in childhood and adolescence [4, 9, 10]. One factor contributing to low rates of treatment is the inadequacy of current systems to identify and connect children and youth with mental health issues to appropriate services [11].

Evidence suggests that universal screening for substance use and mental health disorders should be a routine part of client care in adults [12–16]. However, effective and efficient screening and intervention processes, especially for youth, are only beginning to emerge. At the same time, concerns about unidentified and untreated youth substance use and/or mental health issues have been highlighted across sectors, including child welfare, justice, mental health, addictions, education, health, housing and other social services [17–21]. Accordingly, there is a strong rationale for effective, consistent mental health and substance use screening across youth service delivery settings [12].

Several screening tools are available to identify clinical needs rapidly in community settings. Some of the most popular [12] are the Global Appraisal of Individual Needs-Short Screener [GSS; 22], the Strengths and Difficulties Questionnaire [SDQ; 23], and the Youth Self-Report [YSR; 24] with its parent version Child Behavior Checklist [CBCL; 24]. The SDQ consists of 25 items and screens for internalizing concerns, behavioral and attention concerns, and social strengths and concerns, but not for substance use disorders. The YSR is a longer scale, consisting of 112 items that assess various categories of internalizing and externalizing disorders and thought problems; however, it also does not assess substance use. In contrast, the GSS is appreciated for its very brief format (20 items, 5–7 min to complete) and its design to screen for both substance use and mental health concerns in a single scale. It is validated for use with individuals aged 10 years and older to quickly identify those who may be experiencing various forms of psychosocial difficulties, including substance use problems, and would benefit from a full assessment and treatment planning. These characteristics make it ideal for use in a wide variety of settings, such as outreach and primary care.

The authors (JH, GC) employed the GSS in a Canada-wide collaborative community-based implementation project, the “National Youth Screening Project,” designed to enhance service provider capacity to identify mental health and/or substance use problems in youth aged 12–24 years across clinical (e.g., mental health/addictions) and non-clinical (e.g., education, housing, outreach) sectors [25, 26]. In addition to implementing the use of the screener, the project provided the opportunity to establish a GSS profile of the youth presenting at these sites, while also updating validation data for the GSS and addressing the absence of Canadian youth validation data. It is hypothesized that the GSS will identify high levels of needs among youth and emerging adults across sectors and demonstrate strong psychometric characteristics.

### Objectives

This project has two key objectives: (1) to present profile data for the GSS in a large, national sample of adolescents and emerging adults seeking services in clinical and non-clinical health and social service sectors; (2) to provide incremental evidence of the psychometric properties of the GSS.

## Methods

### Participants

The National Youth Screening Project (NYSP) includes a sample of *N* = 2390 youth aged 12–24 years who presented for service at a participating youth agency within the 6-month project timeframe and participated in the study. Of them, 77 were missing substantial data and were excluded from the analyses, for a final study sample size of *N* = 2313. Youth (and parents/guardians where required) provided consent to share an anonymized copy of these materials used as part of the agency’s services with the NYSP research team. There were 89 participating services representing eight major sectors (child welfare, education, family and social services, health, housing and outreach, justice, mental health, and substance use) across 14 network sites.^1^ All participating services within a network site agreed upon a common 6-month data collection period. Across sites, data collection periods occurred between April 2011 and December 2013. All youth aged 12–24 presenting for service within the data collection period were considered eligible for participation, except those with acute crisis or significant cognitive impairment as determined by the clinician on site, or who had previously completed the GSS within the study timeframe (see ref. 27 for more details).

### Procedure

#### Site processes

Network sites self-selected for participation or were selected by provincial/territorial governments and had to include services from at least three sectors. They were geographically disbursed; included urban, rural and remote settings; and ranged in size from entire provinces or territories (e.g., Prince Edwards Island) to small communities (e.g., Thompson, Manitoba). Networks and project leads met to determine capacity building, research and clinical processes (see ref. [27] for more details).

Service providers were trained using a standardized curriculum to obtain voluntary and informed research consent; administer, score and interpret the GSS for service provision; and use a locally developed referral guide to identify appropriate services, where necessary. Each network site had a coordinator at the lead organization, who served as the local champion for the project. The coordinator was funded by the project to ensure compliance with all project processes and completed training and certification in Ethical Conduct for Research Involving Humans. Research ethics board approval (or organization-specific research review approval) was obtained from all participating organizations, as well as Health Canada and the Centre for Addiction and Mental Health (see ref. [27] for more details).

#### Youth processes

Youth were administered the GSS as part of routine service delivery. With consent, service providers or organizational leads photocopied the research package, ensuring any identifying information was removed prior to submission to the network coordinator and NYSP research team. Voluntary participation and anonymization of data prior to sending to the coordinating site were keys to the ethical conduct of the study. The availability of screening results to guide treatment was a potential benefit to study participants.

### Measures

A one-page background information form was used to gather demographic information about participating youth. It collected information about the determinants of health frequently cited as associated with youth substance use and mental health concerns, including age, sex, education, employment, income support, housing, legal involvement, ethno-racial identification, and language diversity.

An extended version of the GSS was also administered. The GSS is a 20 item self-report screening tool developed by Chestnut Health Systems from the full-length Global Appraisal of Individual Needs-Initial (GAIN-I) [28, 29], which is comprehensive standardized interview protocol that can be used for diagnostic purposes based on DSM-IV-TR symptoms. The GSS presents respondents with a subset of these symptoms, identifying the likelihood of (1) internalizing disorders (e.g., depression); (2) externalizing disorders (e.g., ADHD); (3) substance use problems; and (4) crime and violence. It has been validated in both adults and adolescents, demonstrating strong validity, reliability, specificity, and sensitivity [22]. Chestnut Health Systems permitted project leads from the Centre for Addiction and Mental Health (CAMH): Child, Youth and Family Program to modify the GSS in 2006, by adding seven items to create a 27 item version that was used in this project. The additional items screen for eating-related issues, trauma-related distress, disordered thinking, and gambling, gaming and internet overuse. These supplementary items were added following discussion with members of a multidisciplinary, multi-agency collaborating group identifying gaps in the domains covered by the original GSS. Items were largely based on additional domains covered by the original GAIN-I [28, 29]. The additional items are used in a stand-alone manner to allow clinicians to quickly flag possible areas of concern for further assessment, rather than constituting separate subscreeners. They are therefore not considered to be part of the scale’s factor structure.

The GSS asks participants to indicate the most recent timeframe during which they experienced significant problems in each item area, ranging from never (zero) to past month (3). In the original validation study [22], cut-offs were identified based on the number of items endorsed for the past year per subscreener: 0 items endorsed in a subscreener indicates low likelihood of a need for services, 1–2 items indicates a moderate need, and 3 or higher suggests a high probability of a diagnosis and/or need for services. Responses were recoded as per the scale norms. The GSS total score has a reported 91% sensitivity and 90% specificity at the high probability (3+ items) threshold among adolescents, with internal consistency ranging from α = .65 for crime/violence to α = .81 for externalizing [22]. These past-year cutoffs are used in the current study to characterize the need levels of youth receiving care, based on original validation recommendations.

### Analyses

Descriptive statistics were calculated on demographic variables, GSS subscreeners, and the additional items to describe the sample. Using the established GSS thresholds for a high probability of a need for services, proportions were calculated to examine needs in a service-seeking sample. Twenty-three cases were removed since they did not provide GSS data. Cases missing more than one item per subscreener (>20% of items: *n* = 54; final study *N* = 2313) were also removed from the analyses; the remaining missing cases were handled in a pair-wise deletion manner where appropriate. Child welfare, family and social services, and housing and outreach sectors were collapsed into “Housing, outreach and support” for the purposes of analyses. The GSS item on suicidal ideation was analyzed separately to identify suicidality risk, as a key risk factor to consider for clinicians working with the youth. Descriptive analyses were conducted using SPSS version 21. Internal consistency was calculated using ordinal alphas, through polychoric correlations computed in Stata version 12 following the procedure set out in Zumbo et al. [30].

Confirmatory factor analysis was conducted using EQS 6.2 on the four primary subscreeners of the GSS, with correlated factors. The estimation method used was robust maximum likelihood to account for non-normal distributions and missing data. Recommended cutoffs for fit statistics are as follows: greater than a liberal .90 or a stricter .95 for the non-normed fit index (NNFI) and the comparative fit index (CFI) indicating an acceptable fit, in combination with a cutoff of <.06 for the root mean square error of approximation (RMSEA) [31]. First, a unifactorial model was analyzed. This model did not fit the data. Next, a confirmatory model was produced in accordance with the original report that crime/violence items crossload on the externalizing disorder scale [22]. This model revealed problems, including moderate fit indices and low factor loadings. Additional models were therefore analyzed based on the theoretical model and previous findings to obtain the best-fitting model. Since no acceptable model was found, the results are presented for the model based on the theoretical framework of the scale.

## Results

### Sample description

While the majority of participating youth were from the substance use or mental health sectors, nearly half (44.5%) were from non-clinical sectors (Table 1). The demographic characteristics of participants are presented in Table 2. The majority were White/European, were born in Canada, spoke English as a first language, had completed some high school, and were students or unemployed. The most common age category was 16–18 years. A majority of male youth had been involved with the legal system at some point in their lives.

**Table 1:** Sector distribution of participants

	Male	Female	Total^a,b^
	*n* = 1068	*n* = 1218	*n* = 2286
Addictions	384 (36.0%)	263 (21.6%)	647 (28.3%)
Mental health	212 (19.9%)	409 (33.6%)	621 (27.2%)
Justice	195 (18.3%)	83 (6.8%)	278 (12.2%)
Housing/outreach/support	224 (21.0%)	270 (22.2%)	494 (21.6%)
Education	23 (2.2%)	37 (3.0%)	60 (2.6%)
Health	30 (2.8%)	156 (12.8%)	186 (8.1%)

^a^Participants identifying as “trans” were removed from these analyses due to a small sample size (*n* = 6)

^b^ *N* = 21 cases were missing data for the gender variable and were excluded from the analyses

**Table 2:** Demographic characteristics of the youth who participated

	Male	Female	Total^a,b^
Age—*M* (SD)	17.2 (2.8)	16.6 (2.5)	16.9 (2.6)
12–15 years old—*n* (%)	297 (27.8%)	424 (34.8%)	721 (31.5%)
16–18 years old	485 (45.4%)	559 (45.9%)	1044 (45.7%)
19–24 years old	269 (25.2%)	231 (19.0%)	500 (21.9%)
Missing	17 (1.6%)	4 (0.3%)	21 (0.9%)
Ethnicity—*n* (%)			
White/European	800 (74.9%)	877 (72.0%)	1677 (73.4%)
Aboriginal	92 (8.6%)	123 (10.1%)	215 (9.4%)
Black	20 (1.9%)	43 (3.5%)	63 (2.8%)
Latin American	11 (1.0%)	2 (0.2%)	13 (0.6%)
Asian	4 (0.4%)	4 (0.3%)	8 (0.3%)
Multiple	54 (5.1%)	68 (5.6%)	122 (5.3%)
Other, don’t know	54 (5.1%)	57 (4.7%)	111 (4.9%)
Missing	33 (3.1%)	44 (3.6%)	77 (3.4%)
Highest education—*n* (%)			
Grade 8 or less	176 (16.5%)	231 (19.0%)	407 (17.8%)
Some high school	737 (69.0%)	843 (69.2%)	1580 (69.1%)
High school diploma	94 (8.8%)	94 (7.7%)	188 (8.2%)
Some post-secondary	19 (1.8%)	11 (0.9%)	30 (1.3%)
Post-secondary diploma/certificate	20 (1.9%)	20 (1.6%)	40 (1.7%)
Bachelor’s degree or higher	5 (0.5%)	2 (0.2%)	7 (0.3%)
Other	6 (0.6%)	6 (0.5%)	12 (0.5%)
Missing	11 (1.0%)	11 (0.9%)	22 (1.0%)
Employment status—*n* (%)			
Full time	69 (6.5%)	35 (2.9%)	104 (4.5%)
Part time	124 (11.6%)	118 (9.7%)	242 (10.6%)
Unemployed	274 (25.7%)	268 (22.0%)	542 (23.7%)
Student	515 (48.2%)	718 (58.9%)	1233 (53.9%)
Apprenticeship	4 (0.4%)	2 (0.2%)	6 (0.3%)
Other	17 (1.6%)	21 (1.7%)	38 (1.7%)
Missing/unknown	65 (6.1%)	56 (4.6%)	121 (5.3%)
First language English—*n* (%) yes	985 (92.2%)	1124 (92.3%)	2109 (92.3%)
Missing	36 (3.4%)	30 (2.5%)	66 (2.9%)
Born in Canada—*n* (%) yes	1021 (95.6%)	1163 (95.5%)	2184 (95.5%)
Missing	18 (1.7%)	30 (2.5%)	48 (2.1%)
Unstable/high risk housing—*n* (%) yes	186 (17.4%)	142 (11.7%)	328 (14.3%)
Missing	32 (3.0%)	27 (2.2%)	59 (2.6%)
Legal involvement—*n* (%) yes			
In the last 12 months	458 (42.9%)	219 (18.0%)	677 (29.6%)
More than a year ago	130 (12.2%)	109 (8.9%)	239 (10.5%)
Missing	34 (3.2%)	50 (4.1%)	84 (3.7%)

Sample sizes vary due to missing data

^a^Participants identifying as “trans” were removed from these analyses due to a small sample size (*n* = 6)

^b^ *N* = 21 cases were missing data for the gender variable and were excluded from the analyses

### Descriptive statistics

Table 3 presents mean scores for the four core subscreeners of the GSS, as well as for each item added to the extended version of the scale. Also presented are ordinal alpha scores as indicators of internal consistency for the four subscreeners. All internal consistency scores are within the acceptable range, set by convention at ≥.70 [32]. Ordinal alphas by gender are in a similar range. For females, alphas are .88 for internalizing, .79 for externalizing, .96 for substance use, and .82 for crime/violence. For males, the alphas are .85, .74, .94, and .75 respectively.

**Table 3:** Descriptive statistics and internal consistency by subscreener

	Total^b^			Male^a,b^		Female^a,b^		Male vs. female	Ordinal alpha
	M	SD	Median	M	SD	M	SD	p	
1. Internalizing disorder	1.77	0.88	2.0	1.54	0.88	*1.96*	0.83	<.001	.87
2. Externalizing disorder	1.52	0.73	1.6	1.54	0.72	1.49	0.73	.08	.77
3. Substance problem	1.16	1.00	1.0	*1.32*	0.99	1.01	0.99	<.001	.95
4. Crime/violence	0.79	0.67	0.6	*0.94*	0.68	0.64	0.62	<.001	.80
5. Additional items									–
5a. Missing meals or throwing up	0.62	1.06	0.0	0.28	0.78	*0.90*	1.18	<.001	–
5b. Binge eating	0.68	1.11	0.0	0.35	0.86	*0.96*	1.22	<.001	–
5c. Disturbing memories or dreams	1.49	1.28	2.0	1.21	1.26	*1.72*	1.24	<.001	–
5d. Thinking people are watching you	1.18	1.25	1.0	1.05	1.23	*1.29*	1.25	<.001	–
5e. Seeing or hearing things that no one else could see or hear	0.57	1.04	0.0	0.50	1.00	*0.63*	1.07	.004	–
5f. Videogame playing or internet use	0.67	1.06	0.0	*0.72*	1.07	0.62	1.03	.014	–
5g. Gambling	0.10	0.44	0.0	*0.12*	0.49	0.07	0.37	.009	–
Total score—20 item original version	1.31	0.62	1.25	*1.33*	0.62	1.28	0.62	.022	.89
Total score—27 item extended version	1.16	0.57	1.12	1.15	0.55	1.17	0.58	.224	.90

^a^Participants identifying as “trans” were removed from sex-based analyses due to a small sample size (*n* = 6)

^b^Sample sizes vary due to missing data. Italics indicates the sex group with the significantly higher score, at the *p* < .05 level

### Clinical needs of youth based on the GSS

**Fig. 1:**
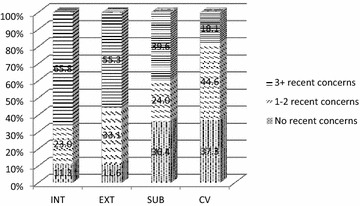
Number of concerns endorsed by GSS domain. This figure shows the percentage of youth endorsing 0, 1–2, or 3+ concerns on each of the four GSS domains. *INT* internalizing domain, *EXT* externalizing domain, *SUB* substance use domain; *CV* crime/violence domain

As can be seen in Fig. 1, nearly two-thirds of participating youth endorsed three or more recent (past 12 months) internalizing concerns, suggesting that with a full diagnostic assessment they would likely meet criteria for a diagnosis in the internalizing disorder domain (e.g. mood disorder, anxiety disorder, etc.). Similarly, in the externalizing disorder domain, over 55% of youth endorsed three or more recent concerns. In the Substance Problem domain, nearly 40% of youth endorsed three or more recent concerns. Endorsement of three or more concerns on the crime/violence subscreener was less common, but almost one-fifth of participants met this threshold. Most youth (81.3%) endorsed three or more recent concerns in at least one of the four domains and would be highly likely to meet criteria for a diagnosis with a full diagnostic assessment.

**Fig. 2:**
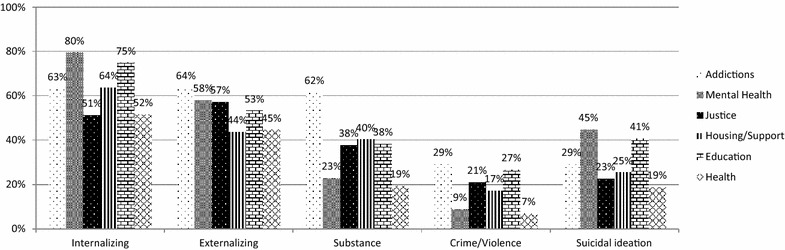
Recent clinical needs using high threshold (3+ endorsements) on GSS domains and past-year endorsement of suicidality, by service sector. This figure represents the proportion of youth endorsing each GSS domain using the high threshold, i.e., endorsing three or more concerns in that domain, and endorsing suicidal ideation. Results are illustrated based on the sector in which the participant entered the study

In Fig. 2, the needs of youth by service sector are presented. In all, 65.8% of youth screened positive for internalizing disorders, 55.3% for externalizing disorders, 39.6% for substance problems, 18.1% for crime/violence, and 31.3% for suicidal ideation. Rates of clinical needs in each domain vary by sector, with statistically significant results for each domain (*p* < .001). In addition to the expected high levels of endorsement in clinical sectors, youth presenting to non-clinical services (i.e., not mental health or addictions) also demonstrated high rates of clinical needs in some domains. Suicidal ideation was endorsed most commonly in the mental health and education sectors, but was present across sectors.

Post-hoc tests at a Bonferroni corrected p value of *p* < .004 reveal that participants who met the criteria in the internalizing domain were significantly more likely to come from the mental health sector (*p* < .001) and less likely to come from the justice (*p* < .001) and health (*p* < .001) sectors. Those meeting externalizing criteria were significantly more likely to come from the addictions sector (*p* < .001) and less likely to come from the housing (*p* < .001) and health sectors (*p* = .002). Participants endorsing substance use disorders were significantly more likely to come from the addictions sector (*p* < .001) and less likely to come from the mental health (*p* < .001) and health sectors (*p* < .001), with the same results for the crime/violence domain. Lastly, those endorsing suicidality were significantly more likely to come from the mental health sector (*p* < .001) and less likely to come from justice (*p* < .001), housing (*p* < .002) or health (*p* = .001).

**Fig. 3:**
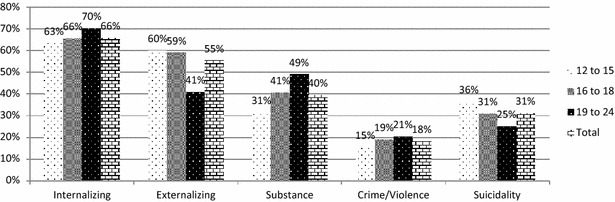
Recent clinical needs using high threshold (3+ endorsements) on GSS domains and past-year endorsement of suicidality, by age group. This figure illustrates the proportion of youth endorsing each GSS domain using the high threshold, i.e., endorsing three or more concerns in that domain, and endorsing suicidal ideation, based on age category (12–15 years old, 16–18 years old, 19–24 years old)

Figure 3 presents the endorsement of clinical needs by age group. Results show that all domains and suicidality varied significantly by age group: internalizing χ^2^ = 6.653, *p* = .036; externalizing χ^2^ = 53.807; *p* < .001; substance use χ^2^ = 39.997; *p* < .001; crime/violence χ^2^ = 6.093; *p* < .048; and suicidality χ^2^ = 17.677, *p* < .001. Post-hoc analyses reveal that for the internalizing domain, none of the analyses reached the Bonferroni-corrected *p* value threshold of <.008, suggesting equivalence across age groups. In the externalizing domain, the younger two age categories were significantly more likely to meet criteria (*p* = .027 and .001 respectively), while the older age group was less likely to meet criteria (*p* < .001). Substance use concerns were significantly less common in the youngest age group (*p* < .001) and more common in the oldest age group (*p* < .001). Age effects did not reach corrected significance in the crime/violence domain. Suicidality was endorsed significantly more often in the younger age group (*p* < .001) and less often in the oldest age group (*p* < .001).

**Fig. 4:**
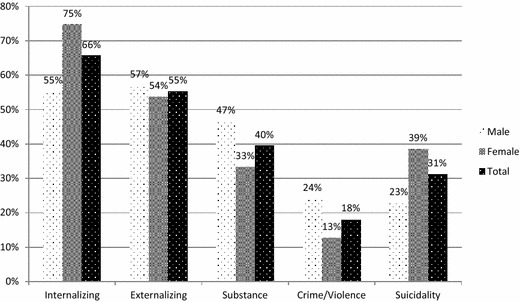
Recent clinical needs using high threshold (3+ endorsements) on GSS domains and past-year endorsement of suicidality, by sex. This figure shows the proportion of youth endorsing each GSS domain using the high threshold, i.e., endorsing three or more concerns in that domain, and endorsing suicidal ideation, based on sex. Participants identifying as “trans” were removed from the analysis due to a small sample size (*n* = 6)

Figure 4 presents subscreener endorsement by sex. Internalizing disorders were most highly endorsed by females (χ^2^ = 95.607, *p* < .001), while substance use concerns (χ^2^ = 41.503, *p* < .001) and crime/violence (χ^2^ = 49.510, *p* < .001) were most highly endorsed by males. The endorsement of externalizing disorders did not differ by sex (χ^2^ = 2.425, *p* = .119). The GSS item regarding suicidal ideation was endorsed by 31.2% of youth, with a significantly higher rate of endorsement among females (χ^2^ = 64.665, *p* < .001).

Subscreener domains were then analyzed by region, defined as Canada’s northerly vs. southerly areas. Results show that internalizing concerns and suicidality were significantly more likely to be endorsed in northerly regions (*p* < .001 and *p* = .032 respectively), but that there was no difference by region for externalizing (*p* = .917), substance use (*p* = .514), or crime/violence (*p* = .775).

**Fig. 5:**
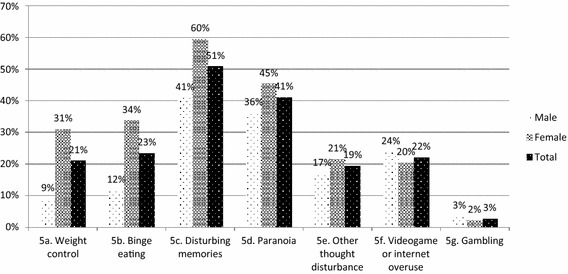
Endorsement of extension items, by sex. This figure shows the proportion of youth endorsing the extension items of the GSS, based on sex. Participants identifying as “trans” were removed from the analysis due to a small sample size (*n* = 6)

Figure 5 presents the endorsement rate of the seven additional items of the extended CAMH version of the GSS, examined in an exploratory manner. Weight control and binge eating concerns were flagged for further assessment by between a quarter and a fifth of youth, with stronger endorsement among females (5a: χ^2^ = 168.325, *p* < .001; 5b: χ^2^ = 155.125, *p* < .001), while disturbing memories or dreams suggestive of trauma-related distress were present in over half of the sample, with higher rates among females (χ^2^ = 78.232, *p* < .001). Although paranoid thoughts were present in over 40% of youth and at a higher rate among females (χ^2^ = 19.669, *p* < .001), other thought disturbances were reported by less than half as many participants, still with higher rates among females (χ^2^ = 7.895, *p* = .005). Videogame/internet concerns were revealed in slightly over one-fifth of youth, with higher rates among males (χ^2^ = 3.901, *p* = .048). Only a small minority of participants endorsed gambling-related concerns, and endorsement did not differ by sex (χ^2^ = 3.295, *p* = .069).

**Fig. 6:**
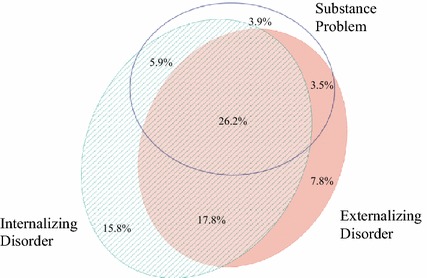
Endorsement of concurrent disorders using high threshold (3+ endorsements). This figure illustrates the proportion of youth with overlapping concerns in the internalizing, externalizing and substance use domains of the GSS, using the high threshold, i.e., endorsing three or more concerns in that domain. 19.0% of respondents did not screen positive in any of the three categories

Overall, 53.5% of youth screened positive for more than one area of concern using the high threshold cutoff value, and this did not differ by sex (χ^2^ = 1.189, *p* = .276). A total of 35.7% of participating youth screened positive for possible concurrent (substance and mental health) disorders. As shown in Fig. 6, 26.2% of all participating youth screened positive for co-occurring internalizing, externalizing and substance use concerns, 5.9% endorsed concurrent internalizing and substance use concerns, and 3.5% endorsed concurrent externalizing and substance use concerns [Venn diagram drawer: 33].

**Fig. 7:**
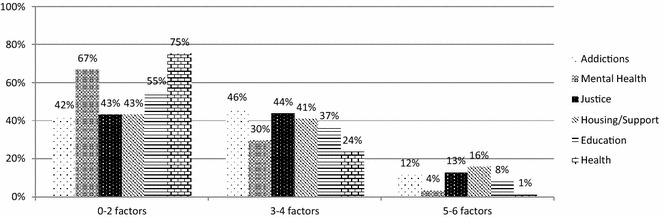
Complexity of needs by service sector. This figure illustrates the proportion of participants endorsing complex needs, based on the service sector in which they entered the study. Complex needs were defined as the endorsement of three or more factors from among (1) housing (unstable/high risk), (2) educational/occupational risk, (3) legal involvement, (4) internalizing disorders, (5) externalizing disorders, and (6) substance use problems

To understand how many participants experience multiple areas of concerns, including sociodemographic risk factors, we examined the proportion of participants endorsing 0–2 factors, 3–4 factors, or 5+ factors based on the service sector at which they presented. Factors examined were housing (unstable/high risk), educational/occupational risk, legal involvement, and internalizing, externalizing, and substance use problems (high-likelihood threshold). Results are shown in Fig. 7.

Notably, over 50% of participants at addictions, housing and justice agencies endorsed three or more factors, with 11.6, 15.8 and 12.9% reporting experiencing five or more of the six factors respectively. Overall, these findings highlight the complexity of the needs of the individuals who are presenting for service, including those presenting in non-clinical service sectors.

### Confirmatory factor analysis

To complement the descriptive findings and add incremental evidence to the psychometric literature on the GSS, confirmatory factor analysis was conducted on the model reflecting the theoretical framework of the scale. The twenty items were distributed evenly on four correlated factors. This model did not fit the data. The Satorra–Bentler Chi square statistic was significant, at SB χ^2^(164) = 2571.3, *p* < .001. The remaining fit statistics were below recommended values: NNFI = .837, CFI = .859, RMSEA = .083 (90% CI .080–.086). The model was rerun with a higher order factor bringing together the four subscales. The higher order factor did not improve the model fit.

To explore the relationship between the extension items and the four subscreeners, the Pearson product-moment correlations between the subscreeners and the extension items of the GSS are presented in Table 4. Weight control, binge eating, disturbing memories, paranoia and other thought disturbance all correlated most strongly with the internalizing subscreener, while videogame or internet use correlated most highly with the externalizing subscreener. Gambling correlated mostly highly, but only weakly, with crime/violence.

**Table 4:** Inter-item correlations between subscreeners and extension items

	1	2	3	4	5a	5b	5c	5d	5e	5f
1. Internalizing										
2. Externalizing	.46									
3. Substance	.30	.42								
4. Crime/violence	.23	.54	.63							
5a. Weight control	.37	.22	.15	.11						
5b. Binge eating	.36	.19	.11	.08	.49					
5c. Disturbing memories	.58	.31	.24	.21	.29	.34				
5d. Paranoia	.45	.31	.20	.22	.26	.29	.42			
5e. Other thought disturbance	.35	.28	.19	.22	.25	.28	.31	.41		
5f. Videogame or internet overuse	.24	.26	.15	.18	.13	.21	.20	.24	.23	
5g. Gambling	.09	.12	.14	.18	.14	.11	.11	.10	.16	.20

All correlations *p* < .01

## Discussion

After successfully training service providers in a wide variety of agency types across Canada to use the GSS to systematically screen for youth addiction and mental health issues, this project examined the rate of problem endorsement in a large sample of Canadian youth presenting for services. Among the youth screened with the GSS, the majority endorsed concerns on the GSS and would be likely to meet diagnostic criteria for a disorder in a full diagnostic assessment, while many endorsed multiple concerns. High clinical need was present in both the clinical and non-clinical sectors. The needs of males and females, and younger and older youth differed. This study also revealed that the GSS’s four-factor structure is not in line with the original study [22] in this sample, suggesting the need for further psychometric work on the scale. However, the utility of the extension items added by the CAMH team was demonstrated; these items may be considered in future revisions of the GSS. In sum, this project demonstrated the feasibility, utility and importance of implementing a systematic screening tool such as the GSS across sectors, while highlighting the need for future psychometric work on the scale.

Previous research shows that the majority of youth with significant substance use or mental health concerns are not connected to specialized clinical treatment services, although they are often connected to other service systems, such as primary care, child welfare or justice [18, 19, 34, 35]. This is partly a result of a lack of adequate problem identification in non-clinical service systems, as well as poor cross-sectoral communication and coordination. For this reason, the current study implemented the GSS screening tool not only in clinically based service settings (e.g., mental health or addictions treatment), but also in non-clinical sectors (e.g., housing, outreach and support), where specialized clinical resources are less common.

A notable finding of the current study was that the rates of severe concerns and complexity endorsed on the GSS were high both among clinically-based services and in non-clinical service agencies (e.g., housing, outreach and support). This result is consistent with service provider perceptions that youth with complex needs often present to non-clinical services without adequate resources to meet their needs [36] and with previous research showing that clinical needs are high across sectors [17–21, 37]. Routine and effective screening for, and assessment of, mental health and substance use concerns among youth across sectors is therefore needed, particularly in service delivery settings where youth are already presenting themselves for assistance. Highlighting this need, many national [38] and international [39] expert panels and organizations have advocated for routine screening for mental health and substance use concerns for youth. Similarly, some youth with significant legal, housing, or vocational needs are seeking services in clinical treatment settings where services tailored to address broader social determinants of health may be less well developed. Taken together, these findings suggest further efforts at collaboration to leverage expertise across sectors and develop effective referral pathways between sectors and agencies may be warranted.

Since over half of participating youth endorsed significant concerns in more than one domain on the GSS, the importance of systematic screening among youth is highlighted [12]. Sex-based differences in individual subscales and items, but not in the GSS total score, illustrate the importance of providing sex/gender-informed services [18–20]. For example, female youth were more likely to endorse internalizing concerns and suicide-related concerns, while male youth were more likely to endorse substance use and crime and violence related concerns. Similarly, youth of different age groups endorsed subscreeners at different levels, highlighting the importance of providing developmentally informed care. About half of youth endorsed having disturbing memories from the past, suggesting the need for trauma-informed care.

The GSS can fill an important gap in identifying youth in need of addiction and mental health services across a broad range of health and social service agency types. Given its acceptability among service providers [25, 26], feasibility of implementation in a broad range of agency types, and ability to screen for both addiction and mental health concerns in a cross-sectoral sample, the GSS can serve a role as an appropriate systematic screening tool for agencies across sectors. However, since its factor structure remains unclear, further work should be conducted to improve the validity of results provided by the scale. This is an important area of work, since systematically screening youth using a brief, cost-effective, user-friendly, and psychometrically valid tool across the full range of health and social service sectors will enable early identification of youth in need of services, potentially facilitating more timely access to needed services. Continued capacity building regarding screening, assessment and treatment of concurrent disorders across health and social service sectors is warranted.

### Limitations

This study has limitations that should be considered when interpreting the results. Since data from a comparable non-service seeking sample were not collected, service-seeking and non-service seeking groups cannot be compared. Given the cross-sectional nature of the study, it is impossible to determine whether the use of the GSS led to the provision of appropriate services. For measure validation purposes, comparison measures were not administered, making it impossible to assess convergence with and discrimination from other measures used in the field. In addition, the “extended” items added to the tool are interpreted on an item-by-item basis rather than forming subscales, which may limit their ability to reliably assess the overall construct that they aim to address; they are intended as general “flags” indicating the need to assess further, rather than fully valid measures of the intended constructs. The reliance solely on self-reports is an additional limitation that may affect the validity and reliability of the results. The problems revealed with the factor structure of the GSS raise questions about the constructs measured by the four domains. Nevertheless, this sample provides a profile of the needs of youth presenting to various health and social service sectors using a popular screening tool that is widely implemented in youth-serving agencies in Canada.

## Conclusions

There have been many calls for a ‘no wrong door’ approach to service delivery for youth in order to improve service engagement, system access and health outcomes [40, 41]. In order for such an approach to be effective, consistent screening with easy-to-implement, valid and reliable tools across multiple sectors is required. By providing a descriptive profile of GSS endorsement among Canadian service-seeking youth, this paper provides benchmarks for those using this screening tool and illustrates the high level of clinical need across health and social service sectors. Moreover, it demonstrates the feasibility and utility of implementing a systematic screening tool and reinforces the need for screening given the high rates of endorsement of multiple areas of concerns by youth across diverse sectors, including those presenting for non-clinical services. The study provides support that the GSS may be a practical tool to assist clinicians in making health service decisions about youth, although further psychometric work is warranted.

## Footnotes

1 (1) Dehcho Region, NWT, (2) Kelowna, BC, (3) Prince George, BC, (4) Thompson, MB, (5) Brantford and Brant County, ON, (6) Haldimand and Norfolk Counties, ON, (7) Niagara Region, ON, (8) Sudbury, ON, (9) Timmins and Region, ON, (10) Ottawa, ON, (11) Prince Edward Island, (12) Pictou County, Cumberland County, and Guysborough/Antigonish/Strait Region, NS, (13) Cape Breton Region, NS, (14) St. John’s, NL.
